# Evaluating ICD-11 anorexia nervosa in a clinical youth sample: a comparison with ICD-10

**DOI:** 10.1007/s00787-026-02972-1

**Published:** 2026-02-25

**Authors:** Luna Thule Viggers, Mie Sedoc Jørgensen, Anne Bryde, Bilal Ashraf, Signe Holm Pedersen, Nadia Micali, Mette Bentz

**Affiliations:** 1 Center for Eating and feeding Disorders Research (CEDaR), Copenhagen University Hospital – Mental Health Services CPH, Copenhagen, Denmark; 2 Child and Adolescent Mental Health Center, Copenhagen University Hospital – Mental Health Services CPH, Copenhagen, Denmark; 3 Great Ormond Street Institute of Child Health, University College London, London, UK

**Keywords:** Anorexia nervosa, ICD-11, Atypical AN, Eating disorders, Symptom severity, Subtypes

## Abstract

Objective: The International Classification of Diseases (ICD) was recently updated to an 11th edition, introducing significant changes to the diagnostic criteria for Anorexia Nervosa (AN). Little is known about how these updated criteria impact diagnostic classification in previously diagnosed populations. This study aims to examine how a Danish youth sample previously diagnosed with ICD-10 AN or Atypical AN (atypAN) would classify under ICD-11 criteria, including the application of the new subtype specifier. Furthermore, it investigates how ICD-11 diagnoses and subtypes relate to ED symptom severity compared to ICD-10 diagnoses. Methods: a prior ICD-10 AN and atypAN sample (N = 895) was reclassified under ICD-11 criteria, categorising those not meeting AN criteria as OSFED. AN cases were subtyped into Restrictive (AN-R) or Binge-Purge (AN-BP). Multiple linear models examined associations between 1) ICD-10 diagnoses; 2) ICD-11 diagnoses; and 3) AN subtypes, and EDE scores, while controlling for age and sex. Results: ICD-11 reclassification led to all ICD-10 AN and 127 atypAN cases meeting AN criteria. Only ICD-11 diagnosis was significantly associated with ED symptom severity, with AN showing greater severity than OSFED. AN-BP exhibited greater severity than AN-R. Discussion: ICD-11 increased number of full-threshold diagnoses and may thus aid in providing adequate treatment, and findings supported the subtyping introduced in ICD-11. However, a substantial amount of prior atypAN cases were not captured in ICD-11 diagnostic criteria.

## Highlights

All prior ICD-10 AN cases met ICD-11 AN criteria, while 127 of 411 prior atypAN cases were reclassified as AN, with the remainder categorized as OSFED.

Only ICD-11 AN diagnosis, not ICD-10 diagnosis, was significantly associated with greater ED symptom severity.

The AN-Binge-Purge subtype exhibited higher levels of ED symptoms than the AN-Restrictive subtype, supporting the subtyping system.

## Introduction

Anorexia Nervosa (AN) is a serious mental disorder frequently emerging in adolescence, carrying significant physical, psychological, and social consequences [1, 2]. Despite being a mental disorder with a relatively low prevalence, increasing rates, including in younger children, are observed [1, 3, 4]. Importantly, early intervention and detection are associated with favourable outcomes [5, 6]. Accurate and inclusive diagnostic criteria are crucial to separate cases from non-cases and enable early identification and intervention. This underpins the purpose of standardized diagnostic systems such as the International Classification of Diseases (ICD) [7–9]. The latest edition, ICD-11, was recently published, replacing ICD-10 [7, 9]. ICD-11 introduced substantial changes to the AN diagnosis, significantly broadening most criteria. For an overview of the changes and differences between ICD-10, ICD-11, and DSM-5 [10], see Table 1.

These changes primarily addressed prior ICD-10 limitations in inclusivity and clinical utility [11]. For instance, ICD-11 removed the requirement for endocrine disturbances (amenorrhea), which was less relevant for males, prepubertal individuals, and other non-typical AN subgroups, despite similar impairment [12]. Moreover, the AN weight criterion has been broadened to include younger individuals, who may instead fail to meet expected growth. Furthermore, the weight criterion can be fulfilled through rapid weight loss, improving recognition of cases across premorbid weight ranges, where previously those of higher premorbid weight would have had to lose significantly more weight to qualify for the diagnosis, despite similar ED symptom severity [13, 14]. This will likely increase the number of individuals meeting the AN criteria.

In addition, ICD-11 removed the requirement of no presence of bulimia and instead introduced subtypes, classifying individuals with AN into Restrictive only (AN-R) or Binge-Purge (AN-BP) subtypes, in line with DSM-5 [9, 10]. However, the clinical significance of these subtypes, especially in child and adolescent samples, remains unclear. Research on the DSM-defined subtypes in adults suggests meaningful differences in both ED symptom severity and outcomes, typically in the direction of AN-BP showing higher severity and worse outcomes [15–17]. However, evidence of this in younger individuals, especially in males, is sparse. Considering the lack of research on ICD-11 subtypes distribution and relation to illness features, such as ED symptom severity in younger samples, such research is warranted as a first step to understanding their potential clinical use.

Additionally, ICD-11 does not include the diagnosis of Atypical AN (atypAN) but places these cases in the residual category of Other Specified Feeding or Eating Disorder (OSFED). This contrasts with its counterpart, DSM-5, where AAN is a sub-category of OSFED, and AAN is met if a patient misses the low weight criterion but meets the remaining diagnostic criteria for AN. By broadening the AN criteria of ICD-11, the hope is that many individuals who previously fell short of full-threshold diagnosis under the ICD-10 may now meet AN criteria. This change likely follows the vast majority of research failing to show that the distinction between atypAN and AN results in significantly different groups in terms of ED symptom severity, course, and outcome, suggesting limited usefulness of the differentiation [12, 18–22].

Removing the atypAN diagnosis and broadening the AN criteria could reduce the need for residual categories such as atypAN, and this aligns with the ICD-11 aim of improving diagnostic inclusivity and reducing residual use. Moreover, the broadening could allow for more accurate differentiation between full-threshold and subthreshold AN in terms of illness features such as ED symptom severity. However, it also prompts important questions about the classification and management of individuals who previously met criteria for atypAN but do not qualify for AN in ICD-11. These individuals are now likely to be diagnosed within the more general OSFED category, which may have implications for both treatment and research. Since younger individuals have often constituted a large proportion of these subthreshold categories [23], it is important to examine the impact of these diagnostic changes in youth populations.

At present, there has been limited research exploring how the ICD-11 AN criteria affect diagnostic distribution in child and adolescent samples and their relationship with ED symptom severity. To our knowledge, the only study available found an increase in full-threshold diagnosis and a reduction of residual category use when employing ICD-11 compared to ICD-10 [11]. However, the study had a small sample with few males, and it did not directly examine the reclassification of atypAN as all restrictive residual categories were analysed as one group. Neither did the mentioned study examine the role of the newly introduced subtypes and their relation to ED symptom severity. Thus, replication in larger samples with larger male representations is warranted. 

### Aims

This study aimed to evaluate the ICD-11 AN criteria in a representative clinical child and adolescent sample previously diagnosed with ICD-10 AN and atypAN. Specifically, it examined how: (1) ICD-11 AN criteria affect diagnostic distribution compared with ICD-10, (2) how cases are distributed across ICD-11 AN subtypes, and (3) how ICD edition and ICD-11 subtypes relate to Eating Disorder Examination (EDE) measured ED symptom severity, while considering the influence of age and sex.

**Table 1:** Main differences in AN and atypical presentation (atypAN/OSFED/AAN) diagnosis between ICD-10 [7, 8], ICD-11 [9], and DSM-5 [10]

Diagnostic criteria	ICD-10	ICD-11	DSM-5
Weight Criterion	- 15% below expected weight.	- Substantially lower than expected weight o Suggest threshold of BMI < 18.5 kg/m2 (adults) or percentile < 5 (children & adolescents) - OR Rapid weight loss ( > = 20% total body weight lost < = six months) - OR failure to meet expected weight (children & adolescents)	- Substantially lower than expected weight - < minimal normal or expected for children.
Endocrine Disturbances (amenorrhea)	- Required	- NA	- Mentioned as possible physical symptom
No Bulimia	- Required	- NA	- NA
Diagnosis for the atypical AN-like presentations	- AtypAN Separate diagnosis - Resembles AN, but may not fulfil one or more criteria	- NA - General OSFED category without subcategories specifying resemblance with AN	- Within the broader OSFED diagnosis, Atypical AN (AAN) is a subtype - All AN-criteria apart from weight > = threshold, despite weight loss
Pattern Specifier	- NA	- Subtypes of AN-BP, AN-R, other defined and undefined. - AN-R = no binge-purging at all - AN-BP = binging and / or purging	- Subtypes of AN-BP and AN-R - AN-R = last 3 months no recurrent binging or purging – primary weight loss method = restriction and/or exercising - AN-BP = last 3 months recurrent binging and purging

## Method

### Design and participants

The study was a naturalistic cross-sectional study. Participants were drawn from a clinical cohort of children and adolescents treated for an ED at a public specialist ED service in Child and Adolescent Mental Health Services (CAMHS) in the Capital Region of Denmark between November 2018 and September 2024. The sample is part of the umbrella research project VIBUS (Effect of Treatment for Children and Adolescents with Eating Disorders), which is initiated by CAMHS and invites all inpatients and outpatients to participate [24, 25]. Consent was obtained by signature from guardian(s) only, when the patient was below 15 years old, and by signature from the patient and their guardian(s) when they were above 15 years old.

Inclusion criteria for the present study were starting treatment in CAMHS, research consent, and a diagnosis of AN (F50.0) or atypAN (F50.1) according to ICD-10, which was still in use in Denmark at the time of inclusion. Exclusion criteria were missing data on Eating Disorder Examination (EDE) Global Scale or Subscales scores. These criteria resulted in a total sample of 895 participants (female *n* = 834, male *n* = 61), see Table 2 for demographics.

**Table 2:** Sample demographics according to ICD-10 and ICD-11 diagnosis

Category	Sample (*N* = 895)	Sex		Age				Height / weight			
		Female	Male	Female		Male		Female		Male	
	*n*	*n*	*n*	*M*	*SD*	*M*	*SD*	*Height M / SD*	*Weight M / SD*	*Height M / SD*	*Weight M / SD*
ICD-10											
AN (F50.0)	484	452	32	14.37	1.70	13.84	2.09	163.9 / 8.8	42.1 / 7.1	165.4 / 10.0	45.1 / 11.0
atypAN (F50.1)	411	382	29	14.49	1.67	14.03	1.98	163.8 / 8.3	45.9 / 8.6	168.3 / 13.1	47.8 / 10.6
ICD-11											
AN	611	577	34	14.47	1.66	13.82	2.14	164.3 / 8.5	43.4 / 7.6	165.2 / 9.8	44.7 / 10.8
OSFED	284	257	27	14.34	1.75	14.07	1.90	162.9 / 8.6	45.0 / 8.8	168.7 / 13.4	48.6 / 10.6

### Context

Project VIBUS overall participation rate is 77% of all treated patients in the capital region. In Denmark, the national guidelines recommend Family-Based Treatment (FBT) as a first-line treatment for AN (F50.0) [26]. FBT is the main treatment offered for AN as well as for atypAN (F50.1) at the site of the study. Despite the diagnosis of Avoidant Restrictive Food Intake Disorder (ARFID) not being included in ICD-10 and first being introduced in ICD-11, the clinic has been diagnosing ARFID using DSM-5 criteria since 2018. Thus, these participants were not part of the current sample, and the atypAN participants should not represent potential ARFID cases.

### Procedures

#### ICD-10 diagnosis

The ICD-10 diagnosis of either AN (F50.0) or atypAN (F50.1) was given prior to the commencement of the current study. All participants had gone through an examination by a multidisciplinary clinical team, including somatic and medical assessments, the Child EDE (ChEDE), and parent and child clinical interview. Subsequently, the information gathered was compiled at a clinical conference with the presence of the consultant psychiatrist where any discrepancies were discussed before determining the final diagnosis.

#### ICD-11 diagnosis

The ICD-11 diagnosis of AN was assigned to all prior ICD-10 AN (F50.0) diagnosed participants. Based on the criteria of the ICD-10 diagnosis of AN (F50.0), it was assumed that participants with this diagnosis would still fulfil the new ICD-11 AN diagnosis. The main difference for these patients is that the weight criterion (Criterion A) has been loosened or can be fulfilled in multiple ways, compared to the weight criterion (A) from ICD-10.

**Fig. 1:**
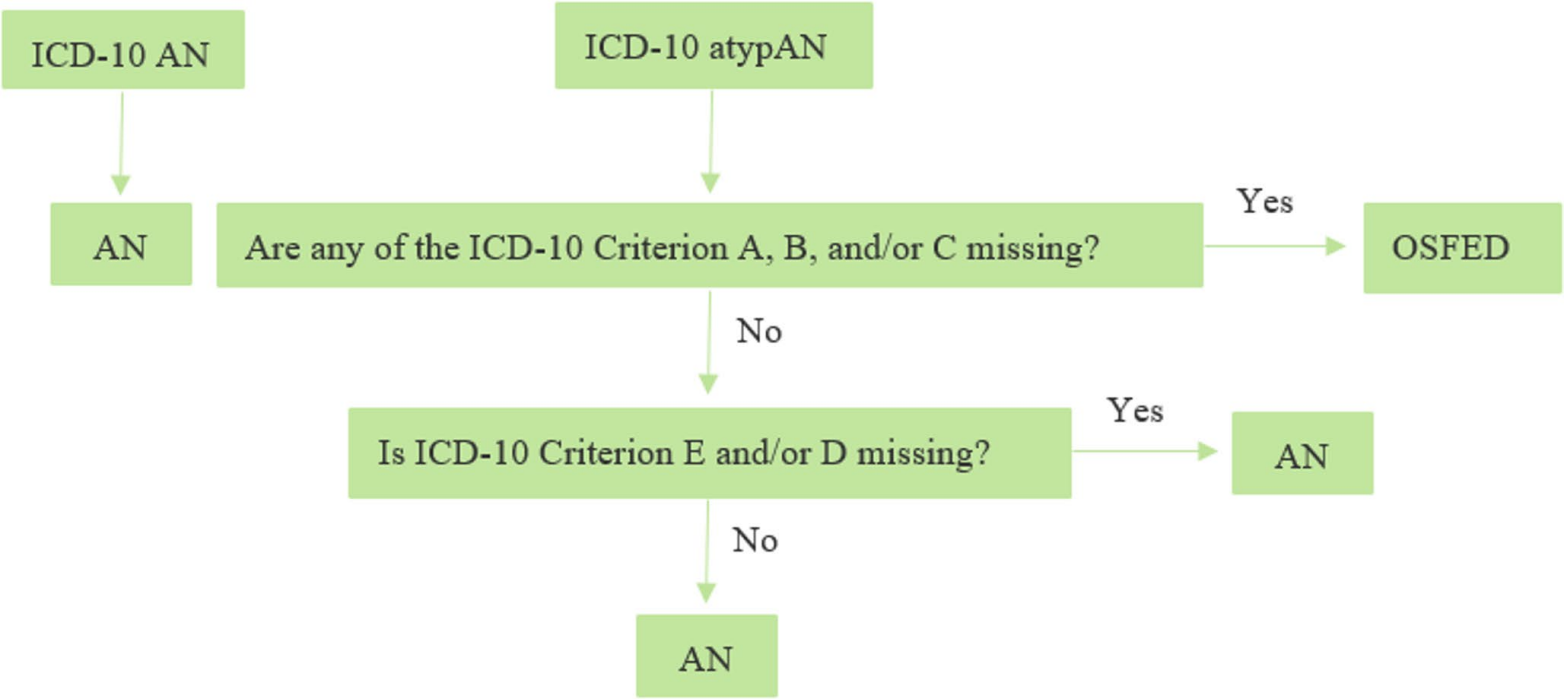
Flow chart showing the diagnostic reclassification into ICD-11 categories of ICD-10 atypAN and AN cases, according to missed ICD-10 criteria

For those diagnosed with ICD-10 atypAN (F50.1), it had been practice in the clinic to record which criterion of AN (F50.0) was not met. These data were used in the current study to decide whether they fulfilled the new AN (ICD-11) diagnosis, as described below and shown in the flowchart in Fig. 1. Participants who did not fulfil ICD-11 AN diagnostic criteria were classified with OSFED. For comparison with other studies we will also report proportion of ICD-11 OSFED who likely would meet DSM-5 criteria for AAN under OSFED.

#### Meeting the ICD-11 AN diagnosis

First, because ICD-11 no longer requires Criterion D (endocrine disturbances) or Criterion E (absence of bulimic behaviours) from the ICD-10 definition of AN, participants who were previously classified as having atypAN *solely* due to not meeting one or both of these criteria were reclassified as having AN under ICD-11. Second, ICD-10 criterion B: *Avoidance of fattening foods*, resembles criterion C in ICD-11, encompassing *restrictive eating or other behaviour associated with maintenance of low weight*. In many ways, the ICD-11 formulation provides a clearer and more comprehensive description of what the ICD-10 criteria B would encompass. Therefore, if failure to meet ICD-10 criterion B was the reason for atypAN classification, the participant would likewise not meet the ICD-11 Criterion C and thus not receive an ICD-11 AN diagnosis. Similarly, the weight criterion of ICD-10 and ICD-11 (criterion A) were assessed to confirm each other, such that participants missing this criterion were not classified with ICD-11 AN. We did not have data to confirm the alternative routes in ICD-11 to fulfil this criterion. Thus, the ICD-10 criterion was relied upon.

Third, if ICD-10 criterion C) *body image distortion with a feeling of being too fat or fear of fatness* was missing, individuals did not fulfil the ICD-11 diagnosis, as this resembles the ICD-11 criterion D. In ICD-11, this criterion is expanded to include behaviours that confirm the presence of excessive preoccupation with body weight or shape. In other words, the patient is no longer required to explicitly state overvaluation of body weight and shape; instead, the clinician can confirm this criterion through parent/teacher/guardian report or direct observation of the individual’s behaviour. However, due to not having clinically assessed the ICD-11 AN symptom in the current study, confirmation of this alternative was not possible.

##### Note

Abbreviations: AN = Anorexia Nervosa, atypAN = Atypical Anorexia Nervosa, OSFED = Otherwise Specified Feeding or Eating Disorder. Criterion A (weight criteria), Criterion B (Avoidance of fattening foods), Criterion C (Body image distortion with a feeling of being too fat or fear of fatness), Criterion D (Endocrine disturbances), Criterion E (Absence of bulimic behaviours).

#### Pattern specifier

Participants were categorised into ICD-11 AN subtypes based on their predominant approach to weight loss and maintenance of low weight. The two main categories are (1) Restricting pattern (AN-R; 6B80.x9) or (2) Binge-Purge pattern (AN-BP; 6B80.x1). When participants were given their initial ICD-10 AN or atypAN diagnosis, it was registered whether there were binge eating and/or purging behaviours according to information from the EDE and/or clinical interview. Since the restricting pattern includes only those who do *not* engage in *any* binge eating or purging behaviours, if binge eating and/or purging were registered, they were categorised as AN-BP (6B80.x1). When none of these behaviours were registered, they were categorised as AN-R (6B80.x0). The unspecified and other specified subtypes were not used as there was no data to support these.

### Measures

#### EDE – children’s version (12th Edition)

The Danish-translated version of the ChEDE 12th edition [27–29] resulted in a global score and four subscale scores, that were used as a dimensional measurement of the severity of ED symptom severity. It was also amongst the tools utilised for determining participants’ initial ICD-10 diagnosis.

Overall, the EDE adult version’s reliability and validity have been supported empirically [28, 30, 31]. Likewise, the ChEDE has shown adequate psychometric properties despite sparse and preliminary research with small samples of wide-ranging ages and often few, if any, male participants [32–34]. Internal consistency of the ChEDE has shown adequate to excellent levels in clinical samples, with Cronbach’s alpha values for the global and subscale scores ranging between 0.65 and 0.95 [32–34], and most items, with a few exceptions, correlating more strongly with their own subscale than others [32, 34]. For the current study, internal consistency estimates were based on available item-level data using pairwise deletion and Cronbach’s Alpha values ranged from moderate to excellent (Eating Concern subscale = 0.64, Restraint subscale = 0.69, Weight Concern subscale = 0.78, Shape Concern subscale = 0.88, Global scale = 0.92).

However, the factor structure, like the adult version [30], has failed to replicate using factor analysis in a German child and adolescent sample [33], though it has been supported in an Australian child and adolescent sample [35]. Thus, though the overall findings suggests that the ChEDE adequately measures ED symptoms, there is uncertainty about the factor structure of the EDE and the ChEDE, indicating that AN symptoms do not naturally cluster together, and these findings question the usefulness of the subscales. Nonetheless, the EDE is the recommended diagnostic tool in research and clinical description of AN-specific symptom severity. Following this, the analysis of the EDE global score is considered the main analysis, and the subscale analyses supplementary.

### Data analysis

The data analyses were carried out in R studio. For aim 1, comparing distribution of diagnoses in the two ICD-editions, and aim 2, describing distribution of ICD-11 subtypes, simple descriptive analyses, or mean and standard deviations (SDs) were used. In addition, a descriptive chi-square analysis examined the diagnostic differences between ICD-10 and ICD-11.

For aim 3, assessing effect of ICD-editions and ICD-11 subtypes on ED symptom severity measured by EDE, two sets of regression analyses were conducted. First, five linear regression analyses were conducted examining the effect of ICD-10 diagnosis (AN vs. atypAN, reference category AN) and ICD-11 diagnosis (AN vs. OSFED, reference category AN) on EDE global score and subscale scores, whilst adding age (continuous) and sex (categorical, reference category Female) as covariates. The models included ICD-10 and ICD-11 diagnosis as main predictors, whilst age and sex were included as covariates. Age and sex were entered as covariates to control for their potential influence. Age was defined as age at diagnosis, and sex was defined as the official registered sex at the time of diagnosis. ICD-10 diagnosis was entered such that positive coefficients indicated higher scores for atypAN than AN. ICD-11 diagnosis was entered such that positive coefficients indicated higher scores for OSFED than AN. Sex was entered such that positive scores indicated higher scores for females than males. These covariates were included as research suggests that adolescents tend to score lower than adults [36], and males tend to score lower than females [37], on the EDE. The primary outcome variable was the EDE Global score, and secondary outcome variables included the subscales of Restraint, Eating Concern, Shape Concern, or Weight Concern Score.

Second, for the subtype analysis, after splitting participants in the ICD-11 AN group according to subtypes (AN-R and AN-BP), we first ran an independent samples t-test with Cohen’s d effect sizes, to compare EDE scores between the two subtypes. Next, five linear regression analyses were used to assess the effect of AN subtype (AN-R vs. AN-BP, reference category AN-BP) on EDE global and subscale scores. These models included AN subtype as the main predictor and age and sex as covariates. The outcome variables were the same as above.

Analyses were tested for the following assumptions for linear regressions: (1) linearity, (2) multicollinearity, (3) normality of residuals, and (4) homoscedasticity. Normality of residuals was violated, but we applied the central limit theorem [38] to the normality of residuals assumption, since all analyses had 500 + data points. To account for heteroscedasticity, Robust Standard Errors (RSE) were applied to all analyses.

## Results

### Diagnostic distribution of ICD-11 AN criteria compared with ICD-10 (aim 1)

Retrospective application of ICD-11 criteria in the sample resulted in 125 females and 2 males previously diagnosed with ICD-10 atypAN (30.9%) to be included in the ICD-11 AN diagnosis. This increased the total number of the sample now meeting ICD-11 AN (*n* = 611, 68.27%) criteria compared with ICD-10 AN (*n* = 484, 54.08%). The remaining 31.73% (*n* = 284) of the sample were categorised with OSFED using ICD-11 criteria compared with 45.92% categorised with atypAN using ICD-10 criteria, indicating a 14.19% decrease in participants in residual categories. A descriptive chi-square test of independence indicated that diagnostic differences between ICD versions were significant, χ^2^(2, *N* = 895) = 709.73, *p* < .001. Table 3 displays the descriptive statistics for each ICD edition and diagnosis by each sex.

Looking at DSM-5 criteria for AAN, 174 (61.27%) of those categorised as OSFED (*n* = 284) in ICD-11 would meet criteria for AAN OSFED in DSM-5. Thus, the DSM-5 AAN diagnosis reduces the number of individuals in general residual categories with additional 19,44% of the total sample, leaving only 12.29% in the general residual OSFED of DSM-5 (compared with 31.73% in ICD-11).

**Table 3:** Descriptive statistics for each EDE score by ICD edition, diagnosis, and sex

EDE Score	ICD-10 Diagnosis				ICD-11 Diagnosis			
	AN (F50.0)		atypAN (F50.1)		AN		OSFED	
	Male (*n* = 32)	Female (*n* = 452)	Male (*n* = 29)	Female (*n* = 382)	Male (*n* = 34)	Female (*n* = 577)	Male (*n* = 27)	Female (*n* = 257)
Global								
*M*	2.56	3.26	2.17	2.85	2.58	3.29	2.11	2.59
*SD*	1.21	1.23	1.55	1.53	1.18	1.22	1.59	1.59
Restraint								
*M*	3.10	3.65	2.71	3.12	3.17	3.64	2.58	2.90
*SD*	1.37	1.38	1.80	1.67	1.37	1.38	1.80	1.76
Eating Concern								
*M*	2.05	2.60	1.63	2.25	2.05	2.63	1.60	2.00
*SD*	1.31	1.27	1.50	1.46	1.27	1.27	1.55	1.47
Shape Concern								
*M*	2.99	3.75	2.40	3.28	3.01	3.79	2.34	2.96
*SD*	1.40	1.47	1.94	1.88	1.36	1.47	2.00	1.98
Weight Concern								
*M*	2.09	3.04	1.95	2.76	2.09	3.09	1.94	2.50
*SD*	1.42	1.61	1.72	1.89	1.37	1.65	1.79	1.86

### Distribution of ICD-11 subtypes (aim 2)

A total of 599 participants fulfilled ICD-11 AN criterion and sufficient information was available to determine their subtype. Participants ranged in age between 9.50 and 17.75 (*M* = 14.43, *SD* = 1.69). Table 4 displays the descriptive statistics of each subtype by sex.

**Table 4:** Descriptive statistics for each EDE score by each ICD-11 subtype and sex

EDE Score	Subtype			
	AN-R		AN-BP	
	Male (*n* = 29)	Female (*n* = 424)	Male (*n* = 5)	Female (*n* = 141)
Global				
*M*	2.43	3.13	3.49	3.86
*SD*	1.06	1.22	1.55	1.00
Restraint				
*M*	3.08	3.48	3.72	4.16
*SD*	1.24	1.37	2.07	1.25
Eating Concern				
*M*	1.90	2.47	2.96	3.21
*SD*	1.19	1.25	1.46	1.13
Shape Concern				
*M*	2.88	3.64	3.77	4.34
*SD*	1.37	1.49	1.18	1.24
Weight Concern				
*M*	1.84	2.91	3.52	3.72
*SD*	1.17	1.62	1.72	1.54

### Influence of ICD-10 and ICD-11 diagnosis on EDE scores (aim 3 part 1)

Five multiple linear regression models examined associations between ICD-10 and ICD-11 diagnosis and EDE global and subscale scores, respectively, whilst adding sex and age as covariates. The models tested the association of each of the independent variables with the ICD10 and ICD-11 diagnosis, after adjusting for the other independent variables. The results of each model are outlined below and can be viewed combined in Table 5.

**Table 5:** *Associations between ICD-11*,* ICD-10 diagnosis*,* age*,* sex and EDE scores: results from linear regression models*

EDE Score	B	SE	t	*p*	*R* ^2^
Global					0.08
ICD-10 atypAN^±^	0.10	0.12	0.82	0.41	
ICD-11 OSFED^±^	− 0.75	0.14	-5.24	< 0.001***	
Age	0.10	0.03	3.63	< 0.001***	
Sex (F)	0.55	0.18	3.05	< 0.01**	
Restraint					0.06
ICD-10	− 0.09	0.14	− 0.66	0.51	
ICD-11	− 0.64	0.16	-3.96	< 0.001***	
Age	0.10	0.03	3.46	< 0.001***	
Sex (F)	0.36	0.21	1.75	0.08	
Eating Concern					0.07
ICD-10	0.12	0.13	0.94	0.35	
ICD-11	− 0.70	0.14	-4.93	< 0.001***	
Age	0.12	0.03	4.61	< 0.001***	
Sex (F)	0.44	0.18	2.40	< 0.05*	
Shape Concern					0.07
ICD-10	0.15	0.15	1.00	0.32	
ICD-11	− 0.93	0.18	-5.26	< 0.001***	
Age	0.10	0.03	2.92	< 0.01**	
Sex (F)	0.66	0.22	2.96	< 0.01**	
Weight Concern					0.04
ICD-10	0.22	0.17	1.27	0.20	
ICD-11	− 0.72	0.19	-3.77	< 0.001***	
Age	0.07	0.03	2.04	< 0.05*	
Sex (F)	0.76	0.21	3.59	< 0.001***	

Note. Abbreviations: F = Female. ^±^: AN is the comparison group, *p < .05. **p < .01. ***p < .001

All models testing the effect of ICD-10, ICD-11, age, and sex on the five different EDE scores were found to be significant (Table 3, *note*). However, *R*^2^ values suggested that somewhat little of the variance was explained by the models, ranging between 4% and 8% of variance explained.

Across all models, atypAN ICD-10 diagnosis was *not* found to have a significant effect on any of the EDE outcome scores when entered alongside ICD-11 diagnosis and after adjusting for the effect of age and sex. In contrast, OSFED ICD-11 diagnosis had a highly significant effect on all EDE outcome scores, where being categorised as OSFED was associated with significantly lower scores across EDE scores compared to AN. Age also showed a significant effect on all EDE scores, where each age unit increase was associated with a slight increase in scores. Sex showed a significant effect on all EDE scores apart from the restraint score, in the direction that being female was associated with a higher score.

### Influence of ICD-11 subtype on EDE scores (aim 3 part 2)

As an initial step, Welch’s independent-samples t-test were performed to compare ICD-11 AN subtypes on EDE scores. These preliminary analyses suggested that AN-R had significantly lower mean scores than AN-BP across EDE scores, with moderate effect sizes: Global, *t*(290.22) = -7.50, *p* < .001, *d* = − 0.68; Restraint, *t*(260.51) = -5.60, *p* < .001, *d* = − 0.52; Eating Concern, *t*(266.90) = -6.90, *p* < .001, *d* = − 0.64; Shape Concern, *t*(290.43) = -5.88, *p* < .001, *d* = -. 53; and Weight Concern, *t*(256.20) = -5.87, *p* < .001, *d* = − 0.55.

To further examine these findings, five linear regression models were run to examine how AN subtype predicted EDE global and subscale scores, respectively, whilst adding sex and age as covariates. The same assumptions as above were violated, and therefore RSE adjusted models, adjusted *R*^2^ values, and Wald’s statistics for model significance are reported. The results of each model are outlined below and displayed combined in Table 6.

**Table 6:** *Regression of associations between subtype*,* age*,* sex*,* and EDE scores*

EDE Score	B	SE	t	*p*	*R* ^2^
Global					0.09
Subtype (R)	− 0.71	0.10	-6.85	< 0.001***	
Age	0.06	0.03	2.19	< 0.05*	
Sex (F)	0.61	0.20	3.08	< 0.01**	
Restraint					0.05
Subtype (R)	− 0.64	0.13	-5.11	< 0.001***	
Age	0.07	0.03	2.15	< 0.05*	
Sex (F)	0.37	0.24	1.55	0.12	
Eating Concern					0.09
Subtype (R)	− 0.71	0.11	-6.25	< 0.001***	
Age	0.09	0.03	2.90	< 0.01**	
Sex (F)	0.47	0.21	2.21	< 0.05*	
Shape Concern					0.06
Subtype (R)	− 0.68	0.13	-5.36	< 0.001***	
Age	0.06	0.04	1.73	0.08	
Sex (F)	0.70	0.24	2.91	< 0.01**	
Weight Concern					0.07
Subtype (R)	− 0.82	0.15	-5.45	< 0.001***	
Age	0.04	0.04	0.99	0.32	
Sex (F)	0.91	0.23	3.92	< 0.001***	

Note. R = Restrictive Subtype; F = Female. *p < .05. **p < .01. ***p < .001

All models testing the effect of subtype on EDE scores whilst adjusting for the effect of age and sex were significant. The *R*^2^ values in these analyses were relatively small, explaining 5–9% of the variance in scores, suggesting that only some of the variance in scores was explained by the models.

Subtype had a significant effect across all models, indicating that being categorised as AN-BP subtype was associated with higher scores on all EDE outcomes. Age had a significant effect in the models of global score, restraint and eating concern where each age unit increase was associated with a 6% higher global score. However, age did not have a significant effect in the model testing shape concern or weight concern. Sex had a significant effect on all EDE outcome score models, apart from the restraint model, in the direction that being female was associated with higher scores. However, due to the small sample of males in these analyses, especially in the binge-purge subtype (*n* = 5), sex findings should be interpreted with caution and considered preliminary.

## Discussion

### Main findings

This study aimed to evaluate the ICD-11 AN criteria in a representative clinical child and adolescent sample previously diagnosed with ICD-10 AN and atypAN, and to examine association between ED symptom severity measured with EDE scores.

Our finding suggested that applying ICD-11 AN criteria increased the proportion of the sample that met full-threshold AN and decreased the proportion categorised as atypical (OSFED) in ICD-11 compared to atypAN in ICD-10. Hence, our findings support the ICD-11 working group’s aim of inclusivity and decreasing residual category use. Despite these efforts, approximately one third of our young sample still fell in the residual category of OSFED in ICD-11, due to either not meeting the weight criterion, the criterion of avoidance of fattening foods, or the criterion of body image distortion. Of note, the subtyping of OSFED in DSM-5 would have categorized more young people as OSFED-AAN, leaving only 12.29% of the sample in the residual non-specific OSFED category. In addition, our findings support the ICD-11 categories being significantly associated with ED symptom severity, in the direction of the AN group scoring higher than the OSFED group, supporting the use of this differentiation. Moreover, the subtype differentiation was significantly associated with ED symptom severity, showing that the AN-BP subtype, on average, displayed higher levels of ED symptom severity.

### ICD edition, inclusivity, and ED symptom severity

The increase in full-threshold AN and decrease in OSFED (vs. atypAN) support the findings of the only previous study examining how the broadening of AN in ICD-11 affected diagnosis in a child and adolescent sample [11]. Nonetheless, in our study, few males were reclassified, suggesting limited ability for the criteria to be inclusive of males, which will need future replication in a sample prospectively diagnosed according to ICD-11 criteria. If it replicates, it might imply that the ICD-11 AN criteria have limited ability to capture male presentations.

Regarding differentiation, prior findings suggested limited differences in ED symptom severity between DSM-5 defined AN and AAN [21, 22]. This is mirrored in our study, where no significant differences in ED symptom severity were observed between ICD-10 AN and atypAN when adjusting for the remaining predictors. By contrast, our findings suggested that the AN broadening in ICD-11 criteria led to more meaningful differences, as AN showed higher ED symptom severity levels than OSFED. Thus, the new criteria may improve some of these prior shortcomings by providing a better clustering of symptom severity. In turn, this could contribute to improved diagnostic accuracy in younger populations, paving the way for timely intervention to aid favourable outcomes.

Nevertheless, a substantial proportion of prior atypAN individuals failed to meet the ICD-11 AN diagnostic threshold and were categorised as OSFED. Interpreting this group as less severe due to their lack of meeting full criteria should be avoided. Their EDE scores, though lower than the current AN group, were still as high or higher than prior child and adolescent AN samples, and substantially higher than the scores of non-clinical samples [32, 34]. Such scores indicate a clinically significant ED in need of treatment. Moreover, the lower scores may reflect a partial inability of the EDE to capture AN presentations in children and adolescents, as research suggests younger individuals are more likely to underreport their symptoms [39, 40]. Suggested reasons for this include unwillingness to report symptoms and lack of cognitive development leading to less insight [40]. Additionally, as noted in the methods section, the new ICD-11 diagnostic criteria were not clinically assessed in this study. Specifically, we were unable to directly evaluate the new ICD-11 weight criterion and in turn relied on participants’ fulfilment of the corresponding ICD-10 criterion, which is not equivalent. Consequently, it is possible that some individuals from the atypAN group classified as ICD-11 OSFED in our study may have met full ICD-11 AN criteria, if a comprehensive clinical assessment had been conducted. Therefore, the findings should be interpreted with caution and require replication and confirmation in fully diagnosed samples.

In addition, in this age group, some individuals may be in developing or early-stage AN, which can result in being categorised as OSFED because they have not yet developed the full symptom presentation. In young individuals, early-stage AN increases the risk for later development of full-threshold AN and has been suggested as a possible age-specific or early-stage reflection rather than true subthreshold cases [23]. Therefore, interpreting this group’s lower EDE scores as indicating less severe and treatment-needing presentations may worsen outcomes and limit early intervention opportunities for this group. Consequently, taking a longitudinal perspective, there is a need for examining this group in terms of differences from the AN group on illness features other than ED symptom severity as well as course and outcome. Such research may highlight whether the group is truly categorically different from AN or whether, in a young population, differences may owe to age representations, developmental differences, or early-illness stages that could still benefit from early detection and intervention.

Lastly, while a proper comparison with DSM-5 is outside the scope of the present study it is worth noting that if ICD-11 had the added sub-category of AAN in OSFED in line with DSM-5 then a subgroup of those not meeting full AN criteria would potentially be more precisely recognised, potentially directing the focus of intervention.

### Subtype and ED symptom severity

Most of our sample meeting ICD-11 AN criteria were classified as AN-R. This somewhat differs from prior DSM subtype-based research in adults showing a more equal split [15, 16, 41]. However, those classified with the AN-BP type are found to be of older age [15], and over time, crossover from AN-R to AN-BP is common [17]. Moreover, binge eating and purging behaviours are less common in younger children [42]. Taken together, this typical course of AN-R being more common in early AN and the relation of AN-BP to older age, likely explains the discrepant finding of our samples subtype distribution to prior findings.

By contrast, our findings support prior findings, showing that the AN-BP subtype, on average, had higher EDE scores than the AN-R subtype across age and sex [15, 16]. The AN-BP subtype higher scores could be due to higher severity or further illness progression. Prior research has both suggested worse outcomes for the AN-BP subtype and found that AN-R is more common in the early illness stages, thus supporting both possibilities [15, 43]. Future research will need to clarify what the severity difference in children and adolescents is indicative of by examining the longitudinal progression, course, and outcome of the ICD-11 subtypes.

### Sex and age

Sex was significantly associated with ED symptom severity in both analyses comparing ICD-editions and subtypes, across all EDE subscales, except for Restraint. This aligns with prior findings that males score lower than females on the EDE [37, 44].

While lower EDE scores in males might suggest lower levels of ED symptom severity, they could also indicate that diagnosis and diagnostic measures are inadequate at capturing male representations of AN. Indeed, there has been evidence of a different, more muscle-focused profile in AN males, with less focus on losing weight [37, 40, 44–46]. Neither the AN diagnosis nor the EDE questions capture this. Hence, future research into sex differences in psychopathological profiles is warranted.

Older age was associated with a higher global score and higher scores on some of the subscales across ICD-edition and subtypes, aligning with prior findings of adults scoring higher than adolescents [36]. This suggests that younger individuals, on average, have lower levels of ED symptom severity, at least on some aspects. However, as discussed, the findings may also reflect limited sensitivity of the EDE and diagnostic criteria to capture younger presentations.

### Strengths and limitations

Our study is the first to directly examine how a large representative clinical ICD-10 AN and atypAN child and adolescent sample was distributed according to ICD-11 AN criteria. However, the retrospective nature of re-classifying the sample with ICD-11 criteria, based on knowledge of prior ICD-10 criteria, limits generalisability and the strength of implications. In particular, the lack of ability of our study to confirm the alternative routes for fulfilling the ICD-11 AN weight criterion (e.g., rapid weight loss or failure to meet expected weight) is a major limitation, as this change is a- if not the most- significant difference between the two ICD versions. In turn, this criterion’s change will likely have a significant impact in practice on fulfilment of the diagnosis. Therefore, our results should be considered with this limitation in mind. For instance, when taking a closer examination of the males in the OSFED groups reasons for failing to meet ICD-11 AN criteria, fifteen missed it only because of and two in part because of criterion A (weight), eight only because of and one in part because of criterion C (body image distortion or fear of fatness) only, and two because of criterion B (avoidance of fattening foods) only. Thus, out of all (*n* = 27) males who were classified as OSFED, a total of seventeen (27.87% of all males) failed to meet the AN criteria, at least in part due to missing the weight criterion, and another nine (5.49% of all males) at least in part due to missing criteria C. Accordingly, our finding that ICD-11 AN criteria may fail to capture males might be less pronounced when ICD-11 is rolled out in clinics and a full diagnostic examination can assess the new criteria. Hence, our findings should be interpreted with caution and studies with the ability for prospective diagnosing with ICD-11 in young samples are needed to validate and replicate our findings. Such research will likely show higher proportions of AN than in the present study, because the alternative routes for the weight criteria and the behavioural manifestations of weight and shape concern can be confirmed. Moreover, despite the relatively large percentage of males, male representativeness in subsamples (e.g., AN-BP) was low, limiting the certainty and generalisability of such findings. Research should continue to include males, aiming to increase our knowledge of this subpopulation. In addition, the findings are based on one clinic in the capital region of Copenhagen, which, as highlighted in the method section, has specific characteristics which may mean that findings will not generalise to other populations. Thus, replication is warranted.

### Implications and future research

Our findings have both clinical and research implications. First, the broader criteria may facilitate earlier and more inclusive AN identification, especially among youth. However, the effect of the broadening for the inclusivity of males is still questionable and needs further clarification. Furthermore, there is a need for future research to clarify how to interpret the “new” OSFED category in terms of severity, outcome, and course, and its differences from AN longitudinally. The findings underline the potential usefulness of considering subtype when planning and designing treatment. Future research should investigate longitudinally how the ICD-11 categories (AN vs. OSFED) and subtypes predict long-term outcomes in a young population, which may increasingly represent early-illness stages, to help guide interventions and address potential changes for the next ICD update.

## Conclusions

This study highlights the potential of ICD-11 criteria to increase the identification of full-threshold AN diagnosis (as opposed to OSFED) and to capture meaningful differences in ED symptom severity within a child and adolescent sample. It further highlights the potential value of AN subtyping in this age group, whilst pointing to the need for further research to clarify differences in ED symptom severity between subtypes. We found that more young persons could be classified with AN using ICD-11 criteria compared with ICD-10 criteria. However, a substantial subgroup was still categorised in the residual diagnostic group despite resemblances with AN, and there may be a risk that their treatment needs are overlooked. However, when a specific diagnostic assessment with ICD-11 AN criteria can take place, more individuals from the prior atypAN group may meet the AN diagnosis than was the case in the current study. Nonetheless, early onset AN is becoming more common and evolving our knowledge of the impact of diagnostic changes on identification, treatment, and outcome remains critical. Our findings provide an initial step towards understanding how the ICD-11 revisions affect diagnostic distribution and their association with ED symptom severity in youth. Future research will need to replicate these findings and clarify their relevance to the course, treatment, and outcome of.

## Data Availability

The data that support the findings of this study may be made available from the corresponding author upon reasonable request. However, restrictions may apply depending on whether data can be sufficiently anonymized at the time of request and may require specific permission from The Danish Data protection Agency.
